# Supplementary material for: Magneto-Luminescent Nanocomposites Based on Carbon Dots and Ferrite with Potential for Bioapplication
Source: Nanomaterials (Basel). 2022 Apr 19;12(9):1396. doi: 10.3390/nano12091396 (PMC9103926; doi:10.3390/nano12091396)
Supplement: Supplementary file 1 [file nanomaterials-12-01396-s001.zip › nanomaterials-1635465-supplementary.pdf]

Supplementary Material

Magneto-Luminescent Nanocomposites Based on Carbon Dots and Ferrite with Potential for Bioapplication

Mariia Stepanova [1,*], Aliaksei Dubavik [1], Arina Efimova [1], Mariya Konovalova [2], Elena Svirshchevskaya [2], Viktor Zakharov [1] and Anna Orlova [1]

[1] International Laboratory Hybrid Nanostructures for Biomedicine, ITMO University, Saint Petersburg 199034, Russia; adubavik@itmo.ru (A.D.); melpomennia@gmail.com (A.E.); vvzakharov@itmo.ru (V.Z.); a.o.orlova@itmo.ru (A.O.)

[2] Department of Immunology, Shemyakin-Ovchinnikov Institute of Bioorganic Chemistry RAS, Moscow 117997, Russia; mariya.v.konovalova@gmail.com (M.K.); esvir@yandex.ru (E.S.)

* Correspondence: mary.s.stepanova@gmail.com; Tel.: +7-(999)-535-45-34

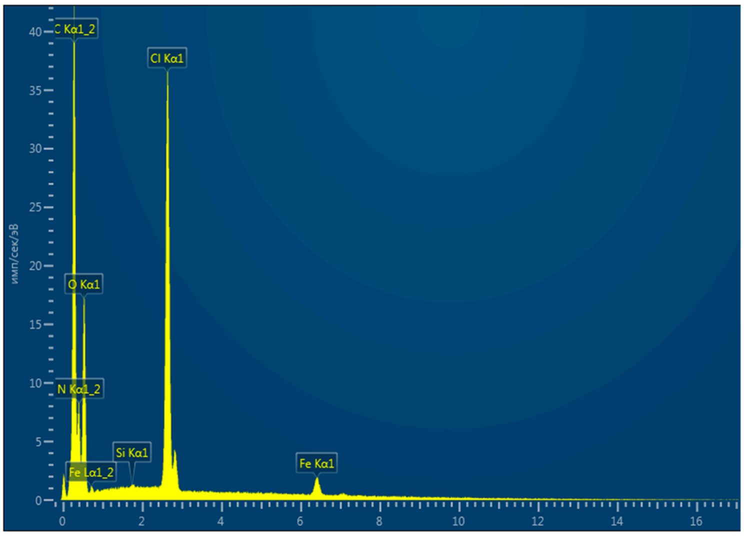

Figure S1. EDX spectra of M-CDs.

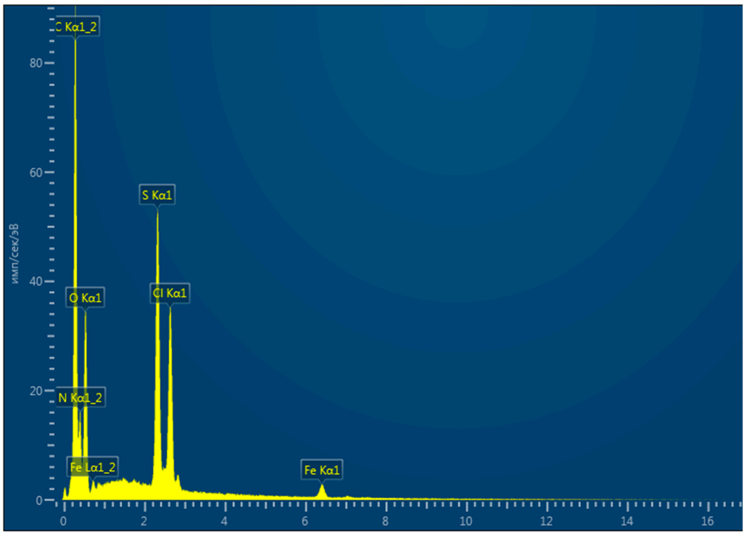

Figure S2. EDX spectra of sM-CDs.

Table S1. Statistics of M-CDs EDX spectrums (atomic percent).

Statistics	C	N	O	Si	Cl	Fe
Max	55.22	23.98	20.17	0.14	3.62	0.41
Min	52.82	21.94	18.75	0.02	2.92	0.30
Averaging	54.13	22.84	19.37		3.26	0.35
Standard deviation	0.73	0.60	0.38		0.19	0.03

Table S2. Statistics of sM-CDs EDX spectrums (atomic percent).

Statistics	C	N	O	Al	Si	S	Cl	Fe
Max	56.04	23.44	19.67	0.03	0.03	2.09	1.61	0.27
Min	54.23	21.42	18.24	0.03	0.02	1.76	1.32	0.22
Averaging	55.22	22.32	18.81			1.91	1.47	0.25
Standard deviation	0.50	0.63	0.49			0.10	0.07	0.01

Table S3. The amplitude-weighted average FL lifetime of M-CDs.

M-CD stock	A_1, kCnts	τ_1, ns	A_2, kCnts	τ_2, ns	$\tau_{av\text{-}Amp}$, ns
water	58 ± 0.6	14.6 ± 0.1	25 ± 0.5	4.5 ± 0.2	11.6 ± 0.1
DMEM	48 ± 0.3	14.2 ± 0.1	21 ± 0.2	4.3 ± 0.1	11.1 ± 0.1

Table S4. The amplitude-weighted average FL lifetime of sM-CDs.

M-CD stabilized	A_1, kCnts	τ_1, ns	A_2, kCnts	τ_2, ns	$\tau_{av\text{-}Amp}$, ns
water	32 ± 0.3	13.3 ± 0.1	14 ± 0.3	4.5 ± 0.1	10.6 ± 0.1
DMEM	29 ± 0.3	13.5 ± 0.1	16 ± 0.3	4.4 ± 0.1	10.3 ± 0.1
